# Integrated single-cell and bulk transcriptomic analyses reveal ferro-aging-related molecular characteristics and immunological heterogeneity in renal cell carcinoma

**DOI:** 10.1371/journal.pone.0362133

**Published:** 2026-11-11

**Authors:** Yang Xu, Yan Zhuang, Qian Fang

**Affiliations:** 1 Department of Urology, Huzhou Central Hospital, Fifth School of Clinical Medicine of Zhejiang Chinese Medical University, Huzhou, China; 2 Department of Urology, Huzhou Central Hospital, Affiliated Central Hospital of Huzhou University, Huzhou, China; 3 Department of Urology, The Affiliated Hospital of Xuzhou Medical University, Xuzhou, China; Shantou University Medical College, CHINA

## Abstract

**Objective:**

Ferro-aging, characterized by chronic iron-dependent oxidative damage and age-related transcriptional alterations, may contribute to the heterogeneity of renal cell carcinoma (RCC). This study aimed to identify ferro-aging-related candidate core genes and evaluate their diagnostic value and biological relevance in RCC.

**Methods:**

GSE40435 and GSE66272 were integrated as the training cohort, and GSE53757 was used for external validation. Ferro-aging-related candidate genes were identified by differential expression and machine-learning analyses to construct a diagnostic model. Their biological relevance was investigated using immune, molecular subtype, WGCNA, single-cell, and virtual knockout analyses, followed by experimental validation of gene expression and LOX function.

**Results:**

A total of 26 ferro-aging-related differentially expressed genes were identified, from which five candidate core genes—DPEP1, LCN2, LOX, NOX4, and TLR4—were selected. The five-gene diagnostic model showed strong discriminatory performance, with AUCs of 0.952 and 0.978 in the training and independent validation cohorts, respectively. These genes were associated with immune-related and molecular features and exhibited heterogeneous cell-type-resolved expression patterns, with the combined five-gene signature showing relatively higher module scores in the epithelial/tumor epithelial compartment. Virtual knockout identified NNMT and FAM134B as potential common response nodes. LOX knockdown significantly inhibited RCC cell proliferation, migration, and invasion and increased lipid peroxidation.

**Conclusion:**

DPEP1, LCN2, LOX, NOX4, and TLR4 represent potential ferro-aging-related diagnostic biomarkers in RCC. LOX may be associated with RCC progression and ferro-aging-related lipid peroxidation, although the underlying mechanisms require further investigation.

## Introduction

Renal cell carcinoma (RCC) is one of the most common malignancies of the urinary system [1,2]. According to recent epidemiological statistics, approximately 435,000 new cases of RCC were reported worldwide in 2022 [3]. Owing to the lack of specific clinical symptoms at the early stage, a considerable proportion of patients are diagnosed with locally advanced disease or distant metastasis, which substantially limits therapeutic options and adversely affects prognosis [4]. In recent years, targeted therapies and immune checkpoint inhibitors have improved the survival outcomes of patients with advanced RCC to some extent [5]. However, marked inter-patient heterogeneity in therapeutic response persists, and drug resistance, recurrence, and disease progression remain major challenges in clinical management [6]. Therefore, the identification of novel biomarkers that reflect the molecular characteristics and progression of RCC is essential for improving early diagnosis and guiding personalized treatment strategies.

Dysregulated iron metabolism and oxidative stress are important biological mechanisms underlying tumorigenesis and tumor progression [7]. Previous studies have demonstrated that ferroptosis, a form of regulated cell death driven by iron-dependent lipid peroxidation, is closely associated with RCC cell survival, tumor progression, and treatment sensitivity [8]. In recent years, the concept of ferro-aging has received increasing attention. Ferro-aging emphasizes the long-term cumulative effects of iron accumulation, lipid peroxidation, oxidative damage, and aging-related transcriptional alterations [9]. Unlike ferroptosis, which mainly focuses on acute iron-dependent cell death, ferro-aging highlights the influence of chronic iron-dependent oxidative injury on cellular function and tissue homeostasis during aging [10]. However, systematic investigations into the expression patterns, diagnostic value, and potential biological functions of ferro-aging-related genes in RCC remain limited.

RCC is characterized by substantial immunological heterogeneity. Its tumor microenvironment consists of diverse immune and stromal cell populations, including T cells, macrophages, monocytes, dendritic cells, endothelial cells, and fibroblasts. These cells play critical roles in tumor immune evasion, inflammatory responses, angiogenesis, and therapeutic response regulation [11–13]. Although immunotherapy has become an important treatment modality for advanced RCC, robust and reliable biomarkers for predicting therapeutic response are still lacking [14,15]. Previous studies have mainly focused on PD-L1 expression, tumor mutational burden, and ferroptosis-related genes [16]. Nevertheless, the relationship between ferro-aging-related molecular features and the immune microenvironment and molecular heterogeneity of RCC has not been systematically elucidated.

With the rapid development of public transcriptomic databases and single-cell RNA sequencing technologies, the integration of bulk transcriptomic data and single-cell-level data has become an effective strategy for dissecting tumor heterogeneity [17]. Bulk transcriptomic analysis enables the identification of disease-associated genes at the population level and facilitates the construction of diagnostic models, whereas single-cell RNA sequencing further reveals the cell-type-resolved distribution, potential cellular origins, and regulatory alterations of candidate genes [18,19]. The combination of these approaches allows a more comprehensive understanding of the biological significance of ferro-aging-related genes in RCC from both global expression patterns and cellular-resolution perspectives.

In this study, we integrated bulk transcriptomic datasets and single-cell RNA sequencing data to systematically investigate the molecular characteristics of ferro-aging-related genes in RCC. We further evaluated their diagnostic value, association with immune heterogeneity, cell-type-resolved distribution, and potential role in tumor progression.

## Materials and methods

### Data collection

Transcriptomic expression datasets related to renal cell carcinoma were retrieved from the Gene Expression Omnibus (GEO) database using the keywords “renal cell carcinoma” and “RCC”. Three datasets, GSE40435, GSE66272, and GSE53757, were ultimately included in the analysis. GSE40435 and GSE66272 were integrated as the training cohort, whereas GSE53757 was used for independent external validation. A total of 128 RCC tissue samples and 128 adjacent normal tissue samples were included in the training cohort. The baseline demographic and clinicopathological characteristics of the 128 patients in the training cohort are summarized in Table 1, and detailed sample-level information is provided in Table S1. Detailed sample information for the external validation cohort GSE53757 is provided in Table S2. Based on the ferro-aging-related gene set reported by Liu et al., 95 ferro-aging-related genes were collected for subsequent analysis (Table S3) [9]. In that study, ferro-aging was defined as an iron-triggered and lipid peroxidation-driven aging program, and the ferro-aging-related gene set was curated by integrating age-upregulated genes with canonical factors involved in iron metabolism, lipid peroxidation, and antioxidant response. In the present study, this previously reported 95-gene set was used as an a priori candidate gene set to explore ferro-aging-related molecular features in RCC. After probe annotation, gene symbol conversion, and data normalization of the raw expression matrices, genes detected in both GSE40435 and GSE66272 were retained. The integrated expression matrix was then corrected for batch effects using the removeBatchEffect() function from the limma package in R software (version 4.2.2), with the dataset source specified as the batch variable. The batch-corrected expression matrix was used for subsequent analyses. Differential expression analysis between RCC tissues and adjacent normal tissues was performed using the limma package. Differentially expressed genes were visualized using volcano plots, box plots, and heatmaps.

**Table 1:** Baseline characteristics of the training cohorts.

Characteristic	GSE40435	GSE66272	Combined cohort
Patients, n	101	27	128
Age, years, mean ± SD	64.12 ± 9.24	64.22 ± 9.19	64.14 ± 9.19
Sex, n (%)			
Male	59 (58.4%)	19 (70.4%)	78 (60.9%)
Female	42 (41.6%)	8 (29.6%)	50 (39.1%)
Tumor stage, n (%)			
Stage I	43 (42.6%)	12 (44.4%)	55 (43.0%)
Stage II	14 (13.9%)	1 (3.7%)	15 (11.7%)
Stage III	11 (10.9%)	13 (48.1%)	24 (18.8%)
Stage IV	14 (13.9%)	1 (3.7%)	15 (11.7%)
Missing	19 (18.8%)	0 (0.0%)	19 (14.8%)
Histological/Fuhrman grade, n (%)			
G1	22 (21.8%)	1 (3.7%)	23 (18.0%)
G2	47 (46.5%)	16 (59.3%)	63 (49.2%)
G3	24 (23.8%)	8 (29.6%)	32 (25.0%)
G4	8 (7.9%)	2 (7.4%)	10 (7.8%)

### Construction of the RCC diagnostic model

Based on the differentially expressed ferro-aging-related genes, feature selection was performed using the support vector machine-recursive feature elimination (SVM-RFE) algorithm. The optimal feature combination was determined through 10-fold cross-validation. Subsequently, the random forest algorithm was applied to evaluate the importance of candidate genes in distinguishing RCC tissues from adjacent normal tissues. Candidate genes were ranked according to the MeanDecreaseGini value. The overlapping genes identified by both algorithms were selected as RCC-related candidate core genes [20]. A multivariable logistic regression model was then constructed based on the expression levels of the candidate core genes. The discriminatory performance of the model was assessed using receiver operating characteristic (ROC) curves and the area under the ROC curve (AUC). ROC curves were generated using the pROC package. In addition, calibration curves were plotted using bootstrap resampling to evaluate the agreement between predicted probabilities and observed outcomes. Decision curve analysis (DCA) was performed to assess the clinical net benefit of the combined diagnostic model and individual gene-based models across different risk thresholds. The fitted five-gene diagnostic model was directly applied to the independent GSE53757 cohort for external validation without model retraining, and its discriminatory performance was evaluated using ROC analysis and the AUC.

### Relationship between candidate core gene expression and the RCC immune microenvironment

The CIBERSORT algorithm was used to estimate the relative proportions of 22 immune cell types based on the batch-corrected expression matrix, with 1,000 permutations performed. The Microenvironment Cell Populations-counter (MCP-counter) algorithm was further applied to evaluate the relative abundance of major immune and stromal cell populations. Spearman correlation analysis was conducted to assess the associations between the expression levels of ferro-aging-related candidate core genes and immune cell infiltration, immune and stromal cell abundance, and inflammation-related molecule expression. Correlation results were visualized using heatmaps. A value of P < 0.05 was considered statistically significant.

### Consensus clustering and comparison of clinical and immunological features

Unsupervised consensus clustering analysis was performed on RCC samples using the ConsensusClusterPlus package based on the expression profiles of the ferro-aging-related candidate core genes. Euclidean distance was used as the distance metric, and repeated resampling was performed to evaluate cluster stability. The optimal number of clusters was determined according to the consensus matrix, cumulative distribution function curves, and cluster stability [21]. The molecular subtyping results were then integrated with clinical information. Patients were stratified by age using the median age as the cutoff value. Categorical variables, including sex, age group, and tumor grade, were compared between molecular subtypes using the chi-square test or Fisher’s exact test, as appropriate. The expression patterns of candidate core genes and the distribution of clinical features across different molecular subtypes were visualized using clinical annotation heatmaps. The MCP-counter algorithm was used to compare differences in immune and stromal cell abundance among molecular subtypes. Differences in inflammation-related molecule expression were also analyzed. Pairwise comparisons were performed using the Wilcoxon rank-sum test, whereas comparisons among multiple groups were performed using the Kruskal-Wallis test. A value of P < 0.05 was considered statistically significant.

### Weighted gene co-expression network analysis (WGCNA) and functional enrichment analysis of key modules

Weighted gene co-expression network analysis was performed to identify co-expression modules associated with RCC molecular subtypes [22]. After standardization of the batch-corrected expression matrix and removal of genes with low expression or low variation, sample and gene quality control was conducted using the goodSamplesGenes function. The optimal soft-thresholding power was determined using the pickSoftThreshold function to construct an approximately scale-free network. A topological overlap matrix was calculated based on the adjacency matrix, and co-expression modules were identified using hierarchical clustering combined with dynamic tree cutting. The minimum module size was set to 50 genes, and the module merging threshold was set to 0.25. Module eigengenes were calculated, and their correlations with RCC molecular subtypes were analyzed to identify key modules. Genes within the key modules were subjected to Gene Ontology (GO) enrichment analysis and Kyoto Encyclopedia of Genes and Genomes (KEGG) pathway enrichment analysis using the clusterProfiler package [23,24]. Adjusted P < 0.05 was considered statistically significant.

### Single-cell RNA sequencing data processing and cell annotation

The RCC single-cell RNA sequencing dataset GSE159115 was obtained from the GEO database. This dataset contains 14 single-cell samples, including RCC tissues and peritumoral tissues. Single-cell data processing and analysis were mainly performed using the Seurat package. A Seurat object was first constructed by retaining genes expressed in at least 10 cells and cells with at least 30 detected genes. Quality control was then performed based on the proportion of mitochondrial genes, and cells with percent.mt < 20% were retained. Environmental RNA contamination was removed using decontX, and potential doublets were identified and excluded using DoubletFinder. After quality control, the data were normalized using NormalizeData. Highly variable genes were identified using FindVariableFeatures, and the data were scaled using ScaleData. Principal component analysis (PCA) was performed using RunPCA. A nearest-neighbor graph was constructed, and cell clustering was performed based on the first 13 principal components. Clustering results at different resolutions ranging from 0.1 to 1.0 were compared, and a resolution of 0.2 was selected because it yielded biologically interpretable major cell populations characterized by distinct canonical marker-gene expression, while avoiding excessive subdivision of transcriptionally similar cell populations observed at higher resolutions. Uniform manifold approximation and projection (UMAP) was used for dimensionality reduction and visualization. Cell types were annotated using the SingleR package with the HumanPrimaryCellAtlasData reference dataset, followed by manual refinement based on classical marker genes. Marker genes for each cell cluster were identified using the FindAllMarkers function, with the criteria of |avg_log2FC| > 1 and adjusted P < 0.05. To evaluate differences in cell-type distribution between RCC tissues and peritumoral tissues, the observed-to-expected ratio (Ro/e) was calculated for each cell type. In addition, the AddModuleScore function was used to calculate a module score for the gene set composed of the five candidate core genes, thereby evaluating the cell-type-resolved distribution of the combined five-gene signature.

### Virtual knockout analysis

Virtual gene knockout analysis was performed using the scTenifoldKnk package. A gene regulatory network was constructed based on the single-cell expression matrix of RCC samples, and highly variable genes were selected for network analysis. Virtual knockout was then simulated for each candidate core gene. Network perturbations following knockout were evaluated based on the differentially regulated genes generated by scTenifoldKnk. Differentially regulated genes with higher perturbation levels were selected for visualization. Significantly differentially regulated genes were identified using an adjusted P-value threshold of < 0.05.

### Cell culture

HK-2, 786-O, ACHN, Caki-1, and A498 cells were obtained from the American Type Culture Collection (ATCC, Manassas, VA, USA). Cells were cultured in the recommended culture medium supplemented with 10% fetal bovine serum and 1% penicillin–streptomycin at 37°C in a humidified atmosphere containing 5% CO_2_.

### Real-time quantitative reverse transcription PCR (qRT-PCR) analysis

Real-time quantitative reverse transcription PCR was performed to measure the expression levels of key genes in normal renal tubular epithelial cells and RCC cell lines, including HK-2, 786-O, ACHN, Caki-1, and A498 cells. Cells in the logarithmic growth phase were collected, and total RNA was extracted using TRIzol reagent. RNA was reverse-transcribed into cDNA according to the manufacturer’s instructions.

Quantitative PCR was performed using SYBR Green qPCR reagent. GAPDH was used as the internal reference gene. Primer sequences are provided in the S4 Table. Relative gene expression levels were calculated using the 2^ − ΔΔCt method.

### Small interfering RNA (siRNA) transfection

Cells were seeded into 6-well plates. When cell confluence reached approximately 50%–70%, LOX-specific siRNA and negative control siRNA were separately transfected into cells according to the instructions of the transfection reagent. Cells were harvested 24–48 hours after transfection, and LOX knockdown efficiency was evaluated by qRT-PCR.

### Cell counting kit-8 (CCK-8) and transwell assays

For the CCK-8 assay, transfected cells were seeded into 96-well plates at a consistent density. CCK-8 reagent was added at predetermined time points, and the cells were incubated according to the manufacturer’s instructions. The optical density at 450 nm was measured using a microplate reader to evaluate cell proliferation. Transwell assays were performed to assess cell migration and invasion. For migration assays, Transwell chambers without Matrigel coating were used. For invasion assays, chambers pre-coated with Matrigel were used. Transfected cells were seeded into the upper chamber, while medium containing serum was added to the lower chamber as a chemoattractant. After incubation for the indicated period, cells remaining on the upper surface of the membrane were removed. Cells that had migrated or invaded through the membrane were fixed, stained, and counted under a microscope in randomly selected fields.

### Lipid peroxidation assay

Lipid peroxidation was assessed by flow cytometry using BODIPY-C11 (Thermo Fisher Scientific, Cat. No. D3861). RCC cells were transfected with LOX-specific siRNA or negative control siRNA. After transfection, the cells were incubated with 5 μM BODIPY-C11 in 2 mL of culture medium at 37°C for 20 min. After washing with phosphate-buffered saline (PBS) to remove excess dye, the cells were resuspended in 500 μL PBS and filtered through a cell strainer before flow cytometric analysis. At least 10,000 cells were analyzed for each sample.

### Statistical analysis

All statistical analyses were performed using R software version 4.2.2. Continuous variables were summarized as mean ± standard deviation (SD), and categorical variables were presented as numbers and percentages. Continuous variables between two groups were compared using Student’s t-test or the Wilcoxon rank-sum test, as appropriate. Comparisons among multiple groups were performed using the Kruskal-Wallis test. Categorical variables were compared using the chi-square test or Fisher’s exact test. Spearman correlation analysis was used to evaluate associations between variables. Differential expression analysis was performed using the limma package. Unless otherwise specified, all statistical tests were two-sided, and P < 0.05 was considered statistically significant.

## Results

### Abnormal expression patterns of ferro-aging-related genes in RCC

Fig 1 illustrates the overall design of the study. Following this workflow, batch effects between the two datasets were first corrected, and the distribution of samples before and after correction is shown in Figs 2A–D. Differential expression analysis was subsequently performed between renal cell carcinoma tissues and adjacent normal tissues to identify differentially expressed genes. Overall, ferro-aging-related genes showed a predominant upregulation trend in tumor samples (Fig 2E). By intersecting the differentially expressed genes with the ferro-aging-related gene set, a total of 26 differentially expressed ferro-aging-related genes were identified, including 20 upregulated genes and 6 downregulated genes (Fig 2F). The expression profiles of these genes are shown in box plots and heatmaps (Figs 2G and H).

**Fig 1:**
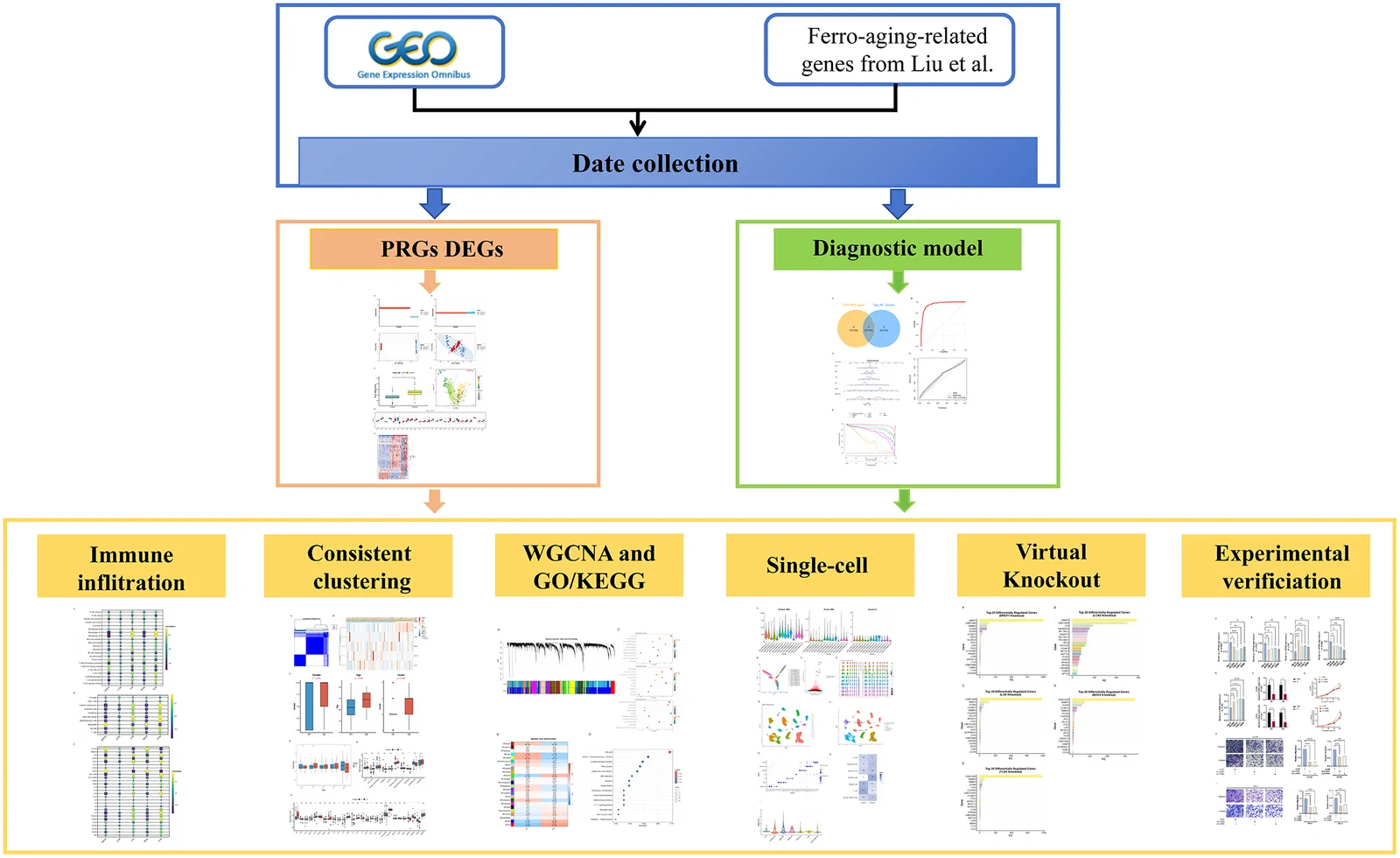
Flow chart of the whole study.

**Fig 2:**
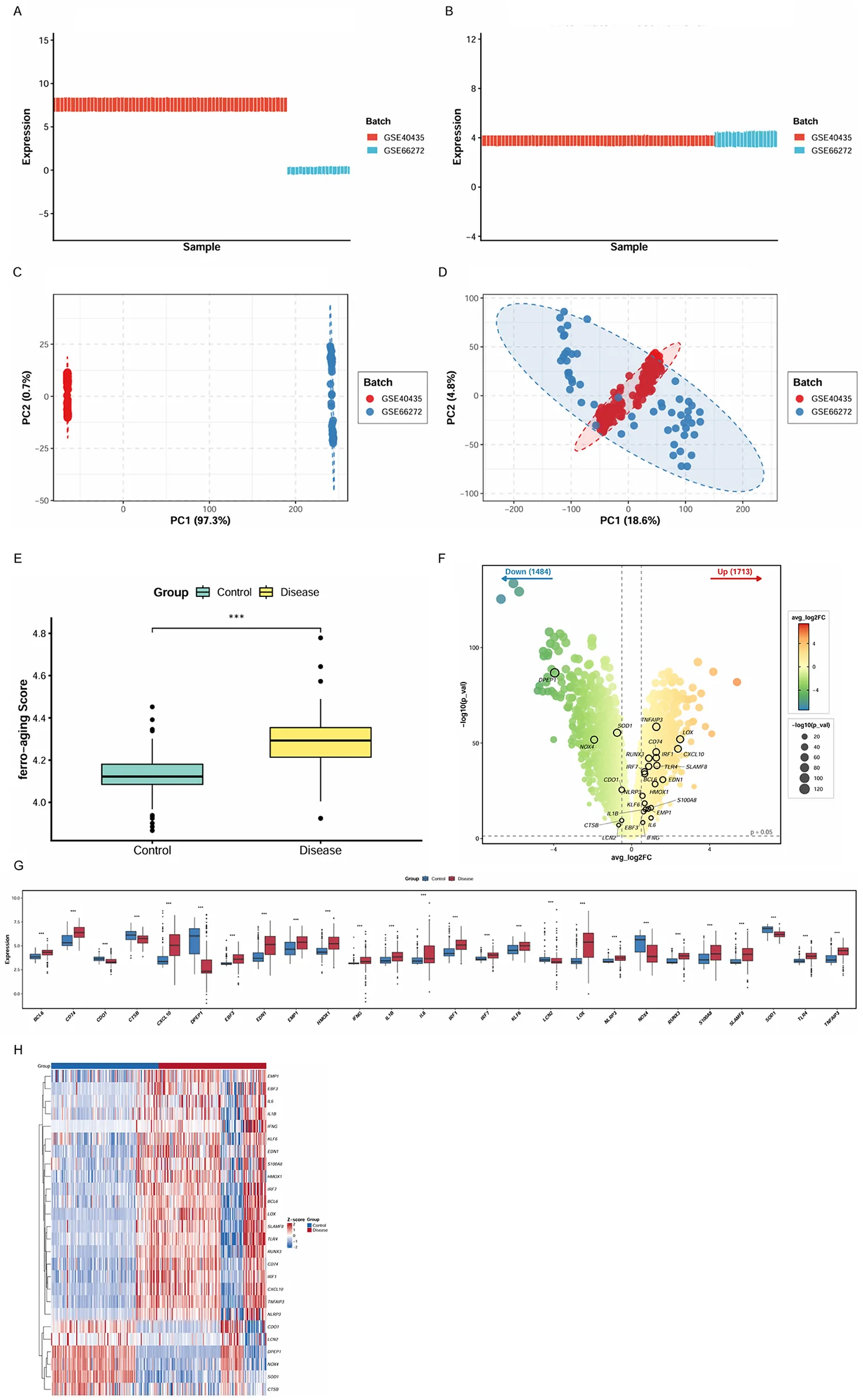
Differential expression of ferro-aging-related genes in renal cell carcinoma. (A) Gene expression distribution before batch effect removal. (B) Gene expression distribution after batch effect removal. (C) PCA plot before batch correction. (D) PCA plot after batch correction. (E) Overall expression levels of ferro-aging-related genes in RCC and adjacent normal tissues. (F) Volcano plot of differentially expressed ferro-aging-related genes. (G) Box plot of differentially expressed ferro-aging-related genes between the two groups. (H) Heat map of differentially expressed ferro-aging-related genes across samples.

### Identification of five ferro-aging-related candidate core genes and construction of a diagnostic model

Feature selection was performed on the 26 differentially expressed ferro-aging-related genes using SVM-RFE and random forest algorithms. Five overlapping candidate core genes, namely DPEP1, LOX, LCN2, TLR4, and NOX4, were identified by the two algorithms (Fig 3A). Because these genes were derived from the predefined ferro-aging-related gene set and were differentially expressed between RCC and adjacent normal tissues, they were considered RCC-associated ferro-aging-related candidate core genes. The diagnostic model constructed based on these five genes showed strong discriminatory performance, with an AUC of 0.952 in the integrated GSE40435 and GSE66272 training cohort and 0.978 in the independent external validation cohort GSE53757 (Figs 3B and S1, respectively). To further visualize the diagnostic contribution of the five candidate core genes, their expression levels were integrated to generate individual RCC risk scores, which were visualized using a nomogram (Fig 3C). The calibration curve showed good agreement between the predicted and observed probabilities (Fig 3D). DCA further indicated that the nomogram model provided a high net benefit across a broad range of risk thresholds and outperformed single-gene models (Fig 3E).

**Fig 3:**
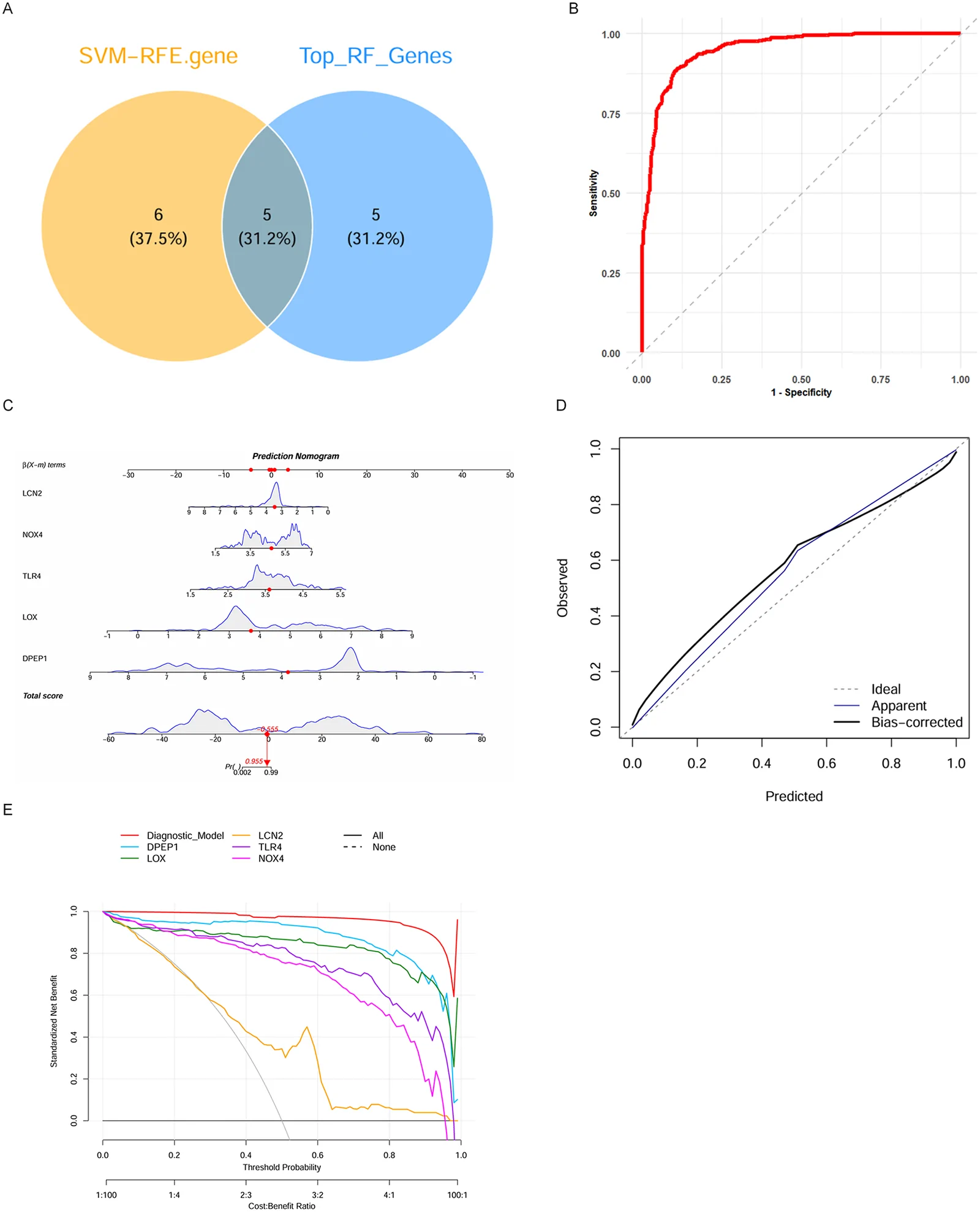
Identification of ferro-aging-related candidate core genes and construction of a diagnostic model. (A) Venn diagram of candidate core genes identified by the SVM-RFE and random forest algorithms. (B) ROC curve of the combined diagnostic model based on the five candidate core genes. (C) Nomogram for RCC diagnosis constructed using the five candidate core genes. (D) Calibration curve of the nomogram model. (E) DCA comparing the clinical net benefit of the nomogram model with that of individual gene models.

### Candidate core gene expression is closely associated with immune infiltration and inflammatory features

To evaluate the relationship between the five candidate core genes and the RCC immune microenvironment, CIBERSORT and MCP-counter analyses were performed. CIBERSORT analysis showed that the expression levels of DPEP1, LCN2, LOX, NOX4, and TLR4 were associated with the infiltration of multiple immune cell types. Among these genes, LOX and TLR4 showed similar correlation patterns and were positively correlated with the infiltration of macrophages, monocytes, CD8 ⁺ T cells, and activated CD4 ⁺ memory T cells. In contrast, DPEP1 and NOX4 generally showed opposite correlation trends (Fig 4A).

**Fig 4:**
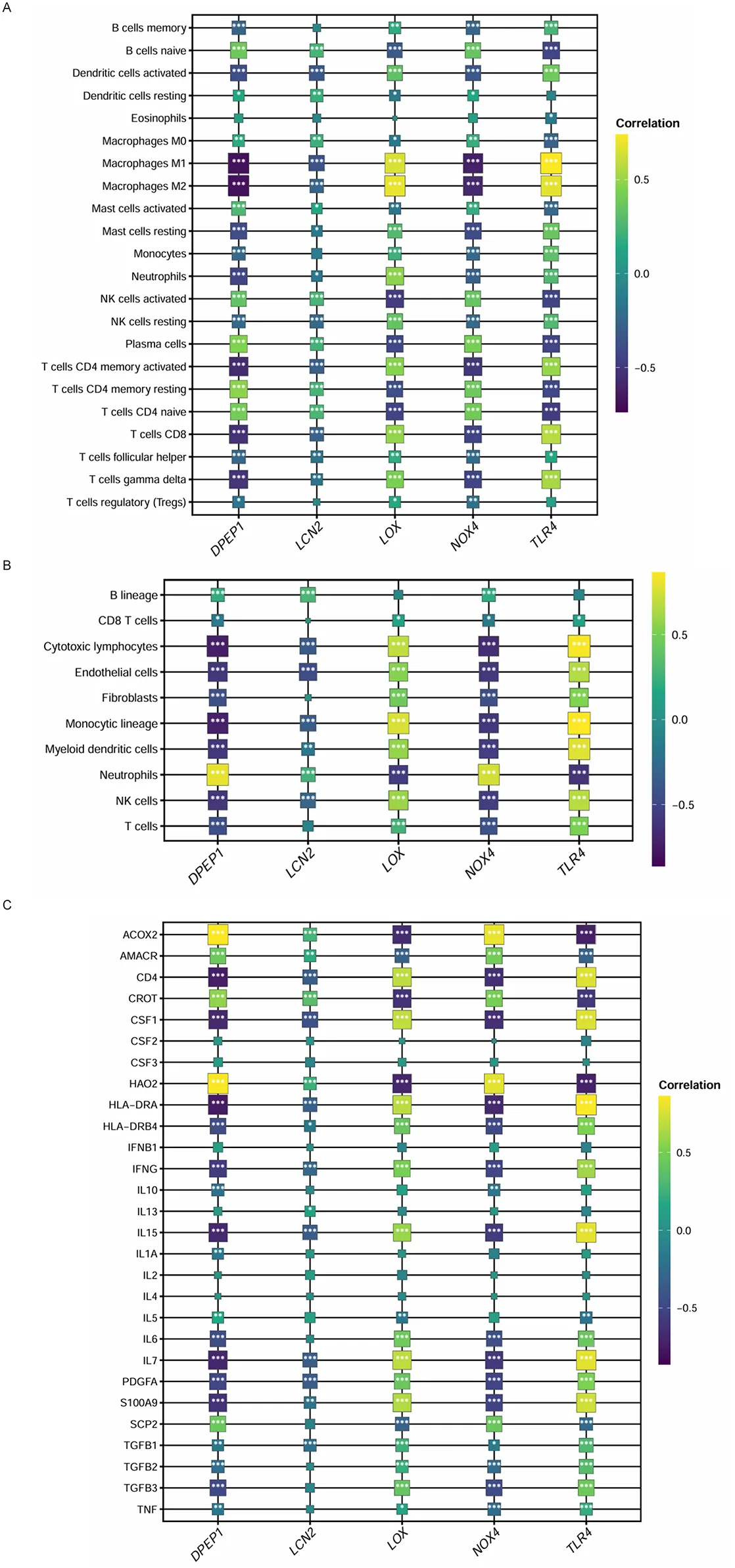
Immune correlation analysis of the five ferro-aging-related candidate core genes. (A) Correlations between candidate core gene expression and 22 immune cell types estimated by CIBERSORT. (B) Correlations between candidate core gene expression and nine immune cell populations estimated by MCP-counter. (C) Correlations between candidate core gene expression and 24 immune- and inflammation-related molecules.

MCP-counter analysis further revealed that LOX and TLR4 were positively correlated with the abundance of cytotoxic lymphocytes, monocyte lineage cells, myeloid dendritic cells, natural killer cells, T cells, endothelial cells, and fibroblasts, whereas DPEP1 and NOX4 were predominantly negatively correlated with these cell populations (Fig 4B). Correlation analysis further revealed distinct associations between the five candidate core genes and immune- and inflammation-related molecules (Fig 4C and S2 Fig). Notably, TLR4 expression was strongly positively correlated with HLA-DRA (r = 0.86), IL15 (r = 0.79), and IL7 (r = 0.79), whereas DPEP1 expression was strongly negatively correlated with HLA-DRA (r = −0.75) and CD4 (r = −0.73). These findings suggest that LOX and TLR4 are associated with an immune-inflammatory microenvironment in RCC, whereas DPEP1 and NOX4 exhibit distinct patterns of association with immune-related features.

### Unsupervised clustering reveals clinical and immunological heterogeneity in RCC patients

Consensus clustering analysis based on the expression profiles of the five candidate core genes showed that 128 RCC samples could be stably classified into two molecular subtypes, designated C1 and C2 (Fig 5A). The heatmap showed distinct candidate core gene expression patterns between the two subtypes (Fig 5B). Comparison of clinical characteristics revealed that patients in the C2 subtype were older and had higher tumor grades, whereas no significant difference in sex distribution was observed between the two subtypes (Fig 5C). Differential expression analysis showed that DPEP1 and LOX were upregulated in the C2 subtype, whereas NOX4 was upregulated in the C1 subtype (Fig 5D). Immune feature analysis further revealed increased monocyte lineage infiltration in the C2 subtype, while the C1 subtype exhibited higher levels of neutrophil infiltration (Fig 5E). Moreover, IL6, IL10, IL7, and CSF1 were upregulated in the C2 subtype, whereas IL13, ACOX2, AMACR, and HAO2 were upregulated in the C1 subtype (Fig 5F). These results indicate that the two ferro-aging-related molecular subtypes of RCC exhibit distinct clinical and immunological characteristics.

**Fig 5:**
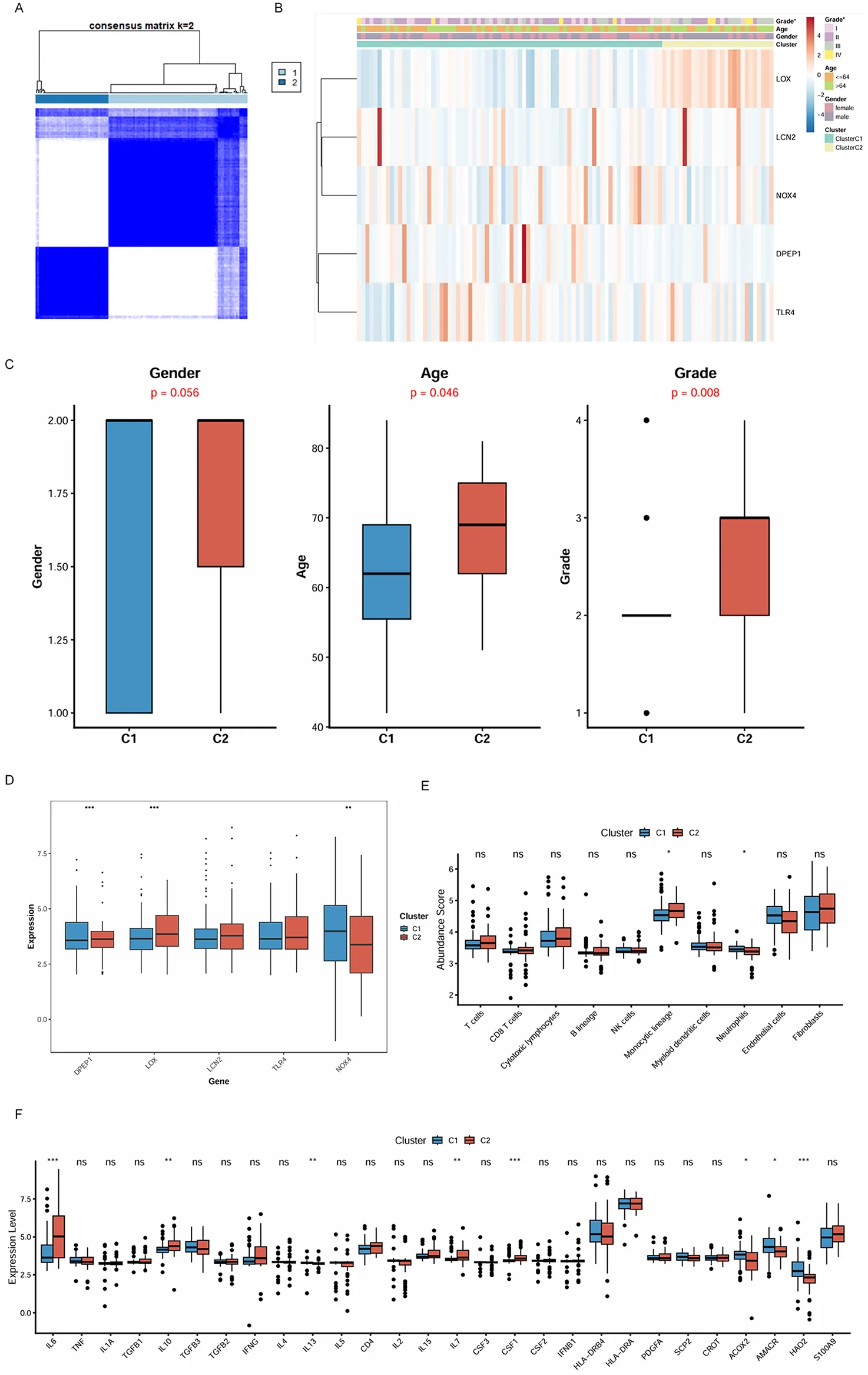
Identification of RCC molecular subtypes by consensus clustering based on the five candidate core genes. (A) Consensus matrix heat map when k = 2; the color gradient from white to blue indicates increasing consensus values among samples. (B) Heat map showing the expression patterns of the five candidate core genes and the distribution of clinical characteristics in the two molecular subtypes. (C) Comparison of sex, age, and tumor grade distributions between the C1 and C2 subtypes. (D) Differential expression of the five candidate core genes between the C1 and C2 subtypes. (E) Differences in the abundance of 10 immune/stromal cell populations between the C1 and C2 subtypes, estimated using MCP-counter. (F) Differential expression of 28 immune- and inflammation-related genes between the C1 and C2 subtypes.

### Identification of RCC-associated co-expression modules and functional enrichment analysis by WGCNA

To further characterize co-expression patterns associated with RCC molecular subtypes, weighted gene co-expression network analysis was performed on RCC samples. Multiple co-expression modules were identified after merging similar modules based on eigengene similarity (Fig 6A). Module-trait correlation analysis showed that the MEred module was negatively correlated with the C1 subtype and positively correlated with the C2 subtype, indicating that it was one of the key modules most closely associated with RCC molecular subtypes (Fig 6B). Functional enrichment analysis revealed that genes in the MEred module were mainly enriched in cell cycle-related biological processes, including cytokinesis, mitosis, and chromosome segregation (Fig 6C). Pathway enrichment analysis similarly showed that this module was significantly involved in proliferation-related pathways, such as the cell cycle and DNA replication (Fig 6D). These findings suggest that the MEred module may represent a key co-expression module associated with cell cycle regulation and tumor cell proliferation in ferro-aging-related RCC molecular subtypes.

**Fig 6:**
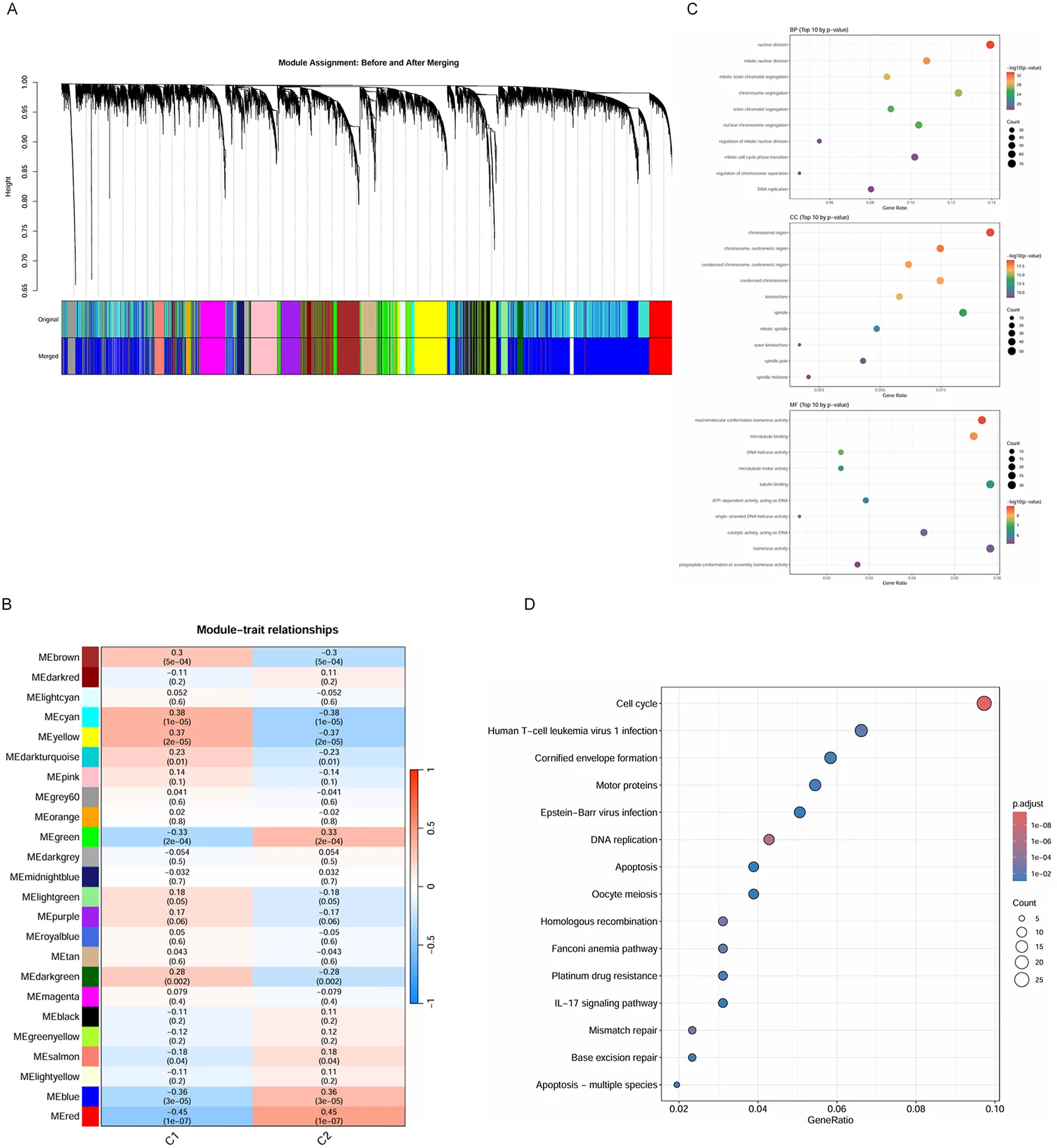
WGCNA identifies a cell cycle-related co-expression module associated with RCC molecular subtypes. (A) Gene clustering dendrogram and module merging results based on the topological overlap matrix. (B) Heat map showing correlations between module eigengenes and the C1/C2 subtypes. (C) GO enrichment analysis of genes in the MEred module. (D) KEGG pathway enrichment analysis of genes in the MEred module.

### Single-cell data integration, clustering, and distribution analysis of the candidate core gene set

After quality control, the number of detected genes, unique molecular identifier counts, and mitochondrial gene proportions remained generally stable across samples (Fig 7A). Following batch effect correction and dimensionality reduction, cells from different sample sources were well mixed in the PCA space, and the top 10 highly variable genes were identified (Figs 7B and C). By comparing clustering results at different resolutions, a resolution of 0.2 was selected for subsequent analysis. Cells were ultimately classified into 18 clusters (Figs 7D and E). Based on marker gene expression and cell type annotation, these clusters were further annotated into seven major cell types, including epithelial/tumor epithelial cells, myeloid cells, T cells, B cells, endothelial cells, vascular/stromal cells, and erythroid cells (Figs 7F and G). To characterize the cell-type-resolved distribution of the five candidate core genes, we examined their expression across major cell populations at single-cell resolution (Figs S3 and S4). The five genes showed heterogeneous expression patterns across cell types. DPEP1 showed prominent expression in epithelial/tumor epithelial cells, whereas TLR4 was more prominently expressed in myeloid cells. LOX and NOX4 showed broader distributions across multiple cellular compartments, with NOX4 also showing appreciable expression in endothelial cells. LCN2 likewise exhibited a heterogeneous distribution across cell populations. Ro/e analysis showed that erythroid cells, endothelial cells, and vascular/stromal-associated cells were relatively enriched in RCC tissues, whereas T cells and myeloid cells were relatively enriched in peritumoral tissues (Fig 7H). Module score analysis further showed relatively higher scores for the combined five-gene signature in epithelial/tumor epithelial cells, whereas relatively lower scores were observed in T cells, myeloid cells, and B cells (Fig 7I). These results indicate heterogeneous cell-type-resolved expression patterns of the individual genes, while the combined five-gene signature shows relatively higher module scores in the epithelial/tumor epithelial compartment.

**Fig 7:**
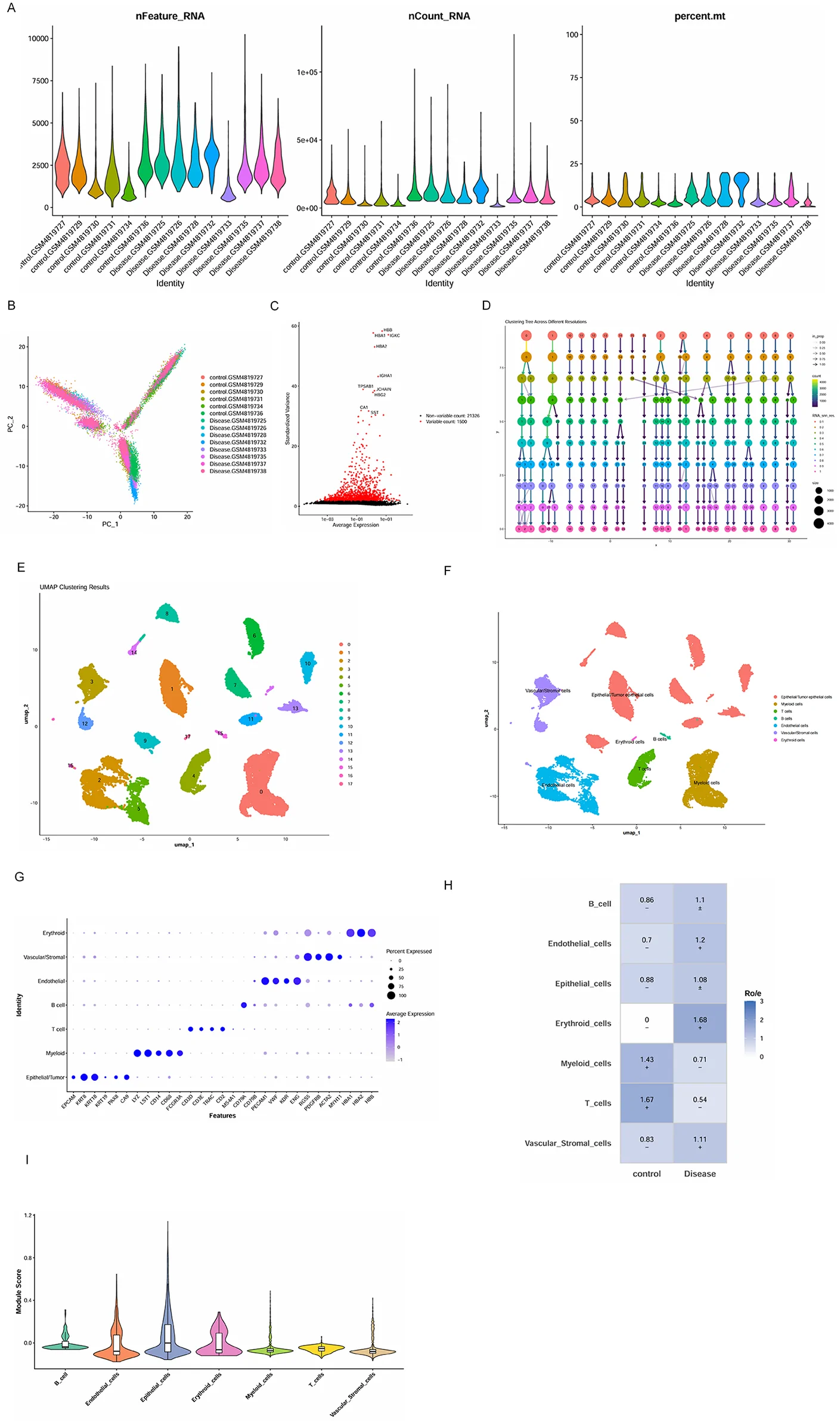
Construction of the RCC single-cell atlas and cell type distribution of the five candidate core genes. (A) Distribution of quality control metrics for single-cell data. (B) PCA plot after batch correction. (C) Top 10 highly variable genes. (D) Cell clustering results at different resolutions. (E) UMAP clustering plot at a resolution of 0.2. (F) UMAP annotation of major cell types. (G) Expression levels of marker genes across cell clusters. (H) Distribution of Ro/e scores for different cell types in RCC and adjacent normal tissues. (I) Module scores of the candidate core gene signature across different cell types.

### scTenifoldKnk virtual knockout analysis predicts candidate-core-gene-associated regulatory network perturbations in RCC

To further explore the influence of the candidate core genes on RCC-related regulatory networks at the single-cell level, virtual knockout analysis was performed using scTenifoldKnk. The results showed that virtual knockout of DPEP1, LCN2, LOX, NOX4, and TLR4 was associated with varying degrees of perturbation in the inferred gene regulatory networks (Fig 8). Virtual knockout of DPEP1 and LCN2 mainly predicted alterations in genes such as NNMT and FAM134B. In contrast, the predicted perturbation patterns after knockout of LOX, NOX4, and TLR4 were relatively consistent, characterized by alterations in FAM134B and shared effects on genes such as NNMT, VCAN, CCND1, SMIM3, and FGG (Figs 8A–E). The recurrent appearance of NNMT and FAM134B across multiple virtual knockout models suggests that they may represent potential common response nodes associated with the RCC candidate core gene regulatory network. Together with the reported functions of the perturbed genes, these virtual knockout results suggest potential links between the candidate core gene-associated regulatory networks and metabolic, proliferative, and microenvironment-related processes in RCC.

**Fig 8:**
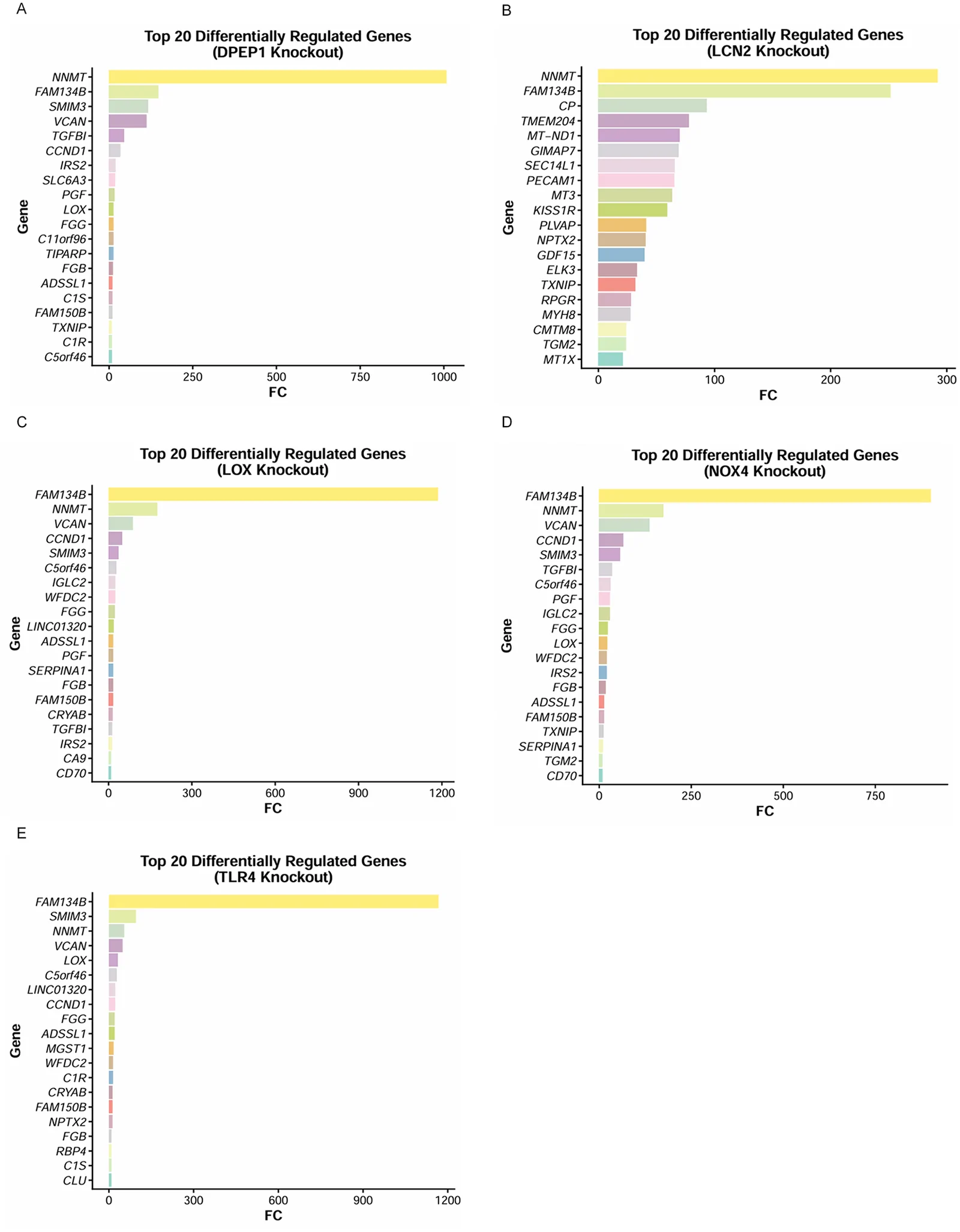
Gene regulatory network perturbation after in silico knockout of the five candidate core genes. (A–E) Top 20 differentially regulated genes after in silico knockout of DPEP1, LCN2, LOX, NOX4, and TLR4, respectively.

### Experimental validation of key genes

To validate the bioinformatics findings, qRT-PCR was performed to detect the expression levels of the five candidate core genes in normal renal tubular epithelial cells and RCC cell lines, including HK-2, ACHN, 786-O, Caki-1, and A498 cells. Compared with HK-2 cells, DPEP1, LCN2, and NOX4 showed an overall trend toward lower expression in RCC cell lines, whereas LOX and TLR4 showed an overall trend toward higher expression (Figs 9A–E). These results further support the aberrant expression of ferro-aging-related candidate core genes in RCC cells. Given the high expression of LOX in the C2 subtype and its potential association with inflammatory infiltration, stromal microenvironment characteristics, and tumor-invasive phenotypes, LOX was selected for further functional validation [25–27]. After siRNA-mediated knockdown of LOX, CCK-8 assays showed a significant reduction in RCC cell proliferation (Figs 9F and G). Transwell assays further demonstrated that LOX knockdown significantly inhibited the migration and invasion abilities of RCC cells (Figs 9H and I). In addition, BODIPY-C11-based flow cytometric analysis showed that LOX knockdown increased lipid peroxidation in RCC cells (S5 Fig).

**Fig 9:**
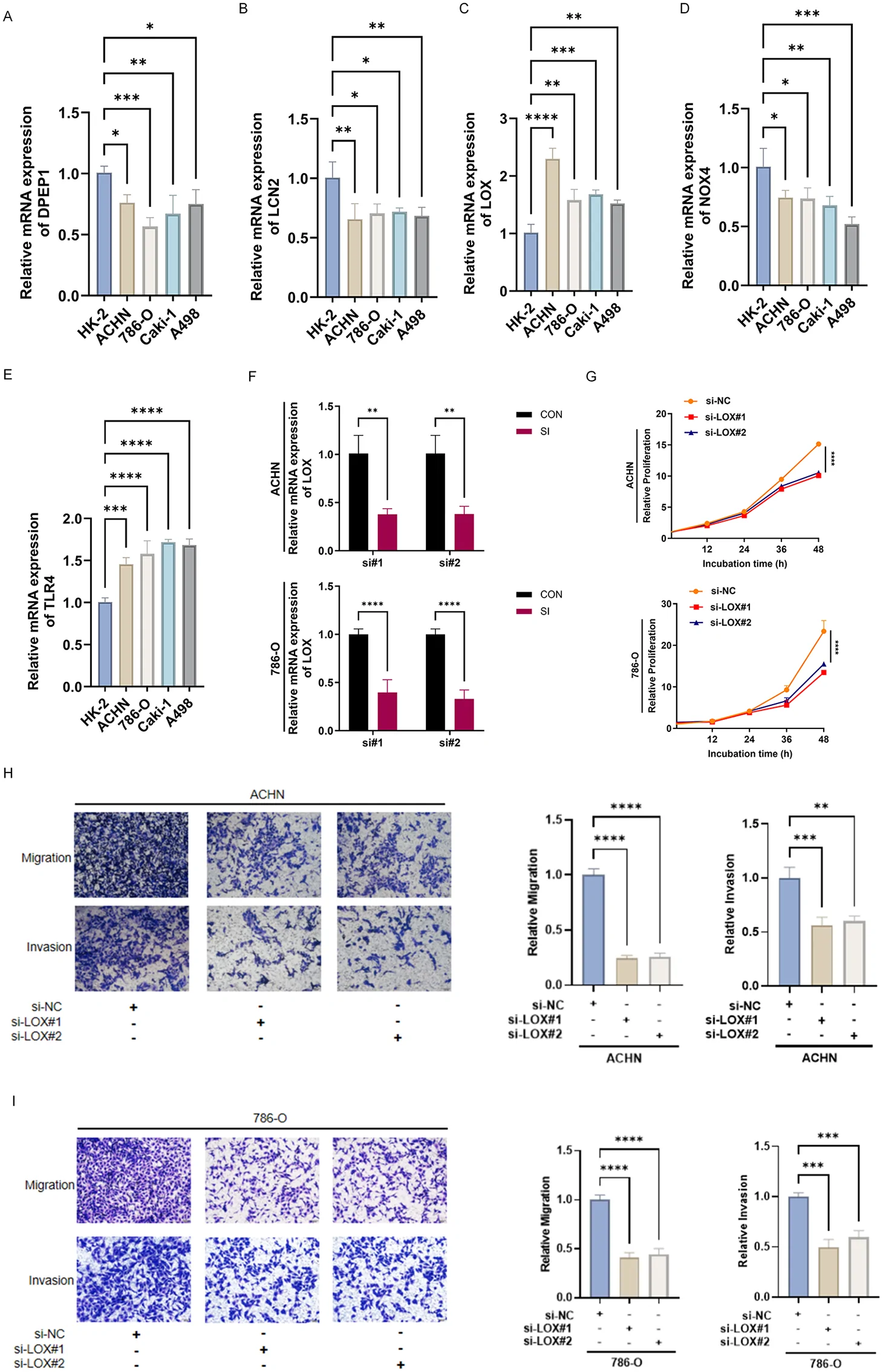
Expression validation of candidate core genes in RCC cell lines and functional analysis of LOX. (A–E) qRT-PCR analysis of DPEP1, LCN2, LOX, NOX4, and TLR4 expression in HK-2 cells and RCC cell lines, including ACHN, 786-O, Caki-1, and A498. (F, G) CCK-8 assays showing the effect of LOX knockdown on RCC cell proliferation. (H, I) Transwell assays showing the effect of LOX knockdown on RCC cell migration and invasion.

## Discussion

This study integrated bulk transcriptomic profiles with single-cell RNA sequencing data to systematically investigate the expression patterns, diagnostic value, and association with immunological heterogeneity of ferro-aging-related genes in renal cell carcinoma (RCC). Through differential expression analysis and machine learning-based feature selection, five ferro-aging-related candidate core genes, namely DPEP1, LCN2, LOX, NOX4, and TLR4, were identified. A multi-gene diagnostic model based on these candidate core genes showed excellent discriminatory performance in distinguishing RCC tissues from adjacent normal tissues in the integrated cohort. Further analyses demonstrated associations of these candidate core genes with immune cell infiltration, inflammation-related molecule expression, and molecular subtype differences. Single-cell analysis further revealed heterogeneous cell-type-resolved expression patterns of the individual genes, with relatively higher module scores for the combined five-gene signature in the epithelial/tumor epithelial compartment. These findings suggest that ferro-aging-related molecular features are associated with RCC biology and tumor microenvironmental characteristics.

Disrupted iron homeostasis, enhanced oxidative stress, and lipid peroxidation accumulation are critical biological events involved in tumorigenesis and tumor progression [28]. Unlike ferroptosis, which mainly refers to acute iron-dependent, lipid peroxidation-mediated regulated cell death, ferro-aging emphasizes the long-term cumulative effects of iron accumulation, oxidative damage, and aging-associated alterations in cellular function [28]. The five candidate core genes identified in this study may reflect different aspects of redox homeostasis remodeling, inflammatory signaling activation, iron-related metabolic alterations, and extracellular matrix remodeling in RCC. Among these genes, LCN2 is closely associated with iron transport and inflammatory responses [29,30]; NOX4 is an important source of reactive oxygen species production [31,32]; TLR4 participates in inflammatory and immune signaling regulation; and LOX is mainly involved in extracellular matrix cross-linking and tissue remodeling [33,34]. The aberrant expression of these genes suggests that ferro-aging-related molecular alterations may be associated with RCC tumor cell phenotypes and tumor microenvironmental features.

The immune microenvironment is a key factor influencing RCC progression and treatment response [35]. In this study, the five ferro-aging-related candidate core genes were found to be associated with immune cell infiltration and the expression of multiple inflammation-related molecules. Specifically, LOX and TLR4 were positively correlated with the abundance of cytotoxic lymphocytes, monocyte lineage cells, myeloid dendritic cells, T cells, endothelial cells, and fibroblasts, as well as with increased expression of several inflammation-related molecules. These findings suggest that LOX and TLR4 are associated with an immune-inflammatory microenvironment, as well as with matrix- and vascular-related features. In contrast, DPEP1 and NOX4 generally exhibited patterns of association with immune-related features, suggesting heterogeneity in the relationships between individual candidate core genes and the RCC immune microenvironment. Further consensus clustering analysis showed that the C2 subtype was characterized by higher tumor grade, increased monocyte lineage infiltration, and elevated expression of multiple inflammation-related molecules. These results suggest that ferro-aging-related molecular signatures may not only have potential diagnostic value but also contribute to the understanding of molecular and immunological heterogeneity among patients with RCC.

WGCNA and single-cell analysis further supported these findings at the pathway and cell-type-resolved levels. WGCNA revealed that the key modules closely associated with ferro-aging-related molecular subtypes were mainly enriched in biological processes such as cell cycle regulation, mitosis, chromosome segregation, and DNA replication. These results suggest that ferro-aging-related molecular characteristics may be linked to proliferative activity in RCC cells. Rapid tumor cell proliferation is usually accompanied by increased iron demand, enhanced oxidative stress, and metabolic reprogramming, while dysregulated iron homeostasis may further affect DNA replication stress, oxidative damage repair, and cell cycle progression [36,37]. Single-cell analysis showed relatively higher module scores for the five-gene signature in epithelial/tumor epithelial cells, whereas lower scores were observed in T cells, B cells, and myeloid cells. This finding indicates that the combined five-gene signature is more prominently represented in the epithelial/tumor epithelial compartment at the transcriptomic level. In addition, the relative enrichment of endothelial cells and vascular/stromal-associated cells in RCC tissues further indicates differences in the cellular composition of the tumor-associated microenvironment.

In this study, scTenifoldKnk was further used to perform virtual knockout analysis and predict potential regulatory network changes after disruption of the candidate core genes. The results showed that virtual knockout of each of the five candidate core genes caused varying degrees of perturbation in gene regulatory networks. Notably, NNMT and FAM134B appeared repeatedly across multiple knockout models, suggesting that they may act as common response nodes following disruption of different candidate core genes. NNMT is associated with metabolic reprogramming and methylation regulation, whereas FAM134B is involved in endoplasmic reticulum autophagy and protein homeostasis [38,39]. These computational findings suggest potential associations between perturbation of the candidate core gene-related networks and metabolic regulation, autophagy homeostasis, and oxidative stress-related processes in RCC. However, scTenifoldKnk is a computational inference method based on single-cell expression matrices [40]. Therefore, its results should be interpreted as hypothesis-generating evidence rather than direct proof of biological causality.

For experimental validation, qRT-PCR confirmed the aberrant expression of the five ferro-aging-related candidate core genes in RCC cell lines. Functional validation was further performed for LOX. The results showed that siRNA-mediated knockdown of LOX significantly inhibited RCC cell proliferation, migration, and invasion, suggesting that LOX may participate in maintaining the malignant phenotype of RCC cells. Furthermore, BODIPY-C11-based flow cytometric analysis showed that LOX knockdown increased lipid peroxidation levels in RCC cells. These experimental findings are consistent with the bioinformatics results, which showed that LOX expression was elevated in the C2 subtype and was closely associated with higher tumor grade, inflammatory infiltration, and stromal microenvironmental characteristics. Previous studies have indicated that LOX not only participates in extracellular matrix remodeling and collagen cross-linking but also affects tumor cell migration, invasion, and tumor microenvironment remodeling [41–43]. Therefore, LOX may be associated with RCC progression through processes related to extracellular matrix remodeling and tumor cell motility, while the present findings additionally suggest an association between LOX and lipid peroxidation. However, because intracellular iron accumulation, ROS production, additional ferroptosis-related markers, and cellular senescence markers were not assessed, the present findings provide only preliminary evidence linking LOX to ferro-aging-related lipid peroxidation and do not establish a direct mechanistic role for LOX in ferro-aging or ferroptosis.

Compared with previous studies that mainly focused on constructing RCC prognostic models based on ferroptosis-related genes, this study focused on ferro-aging, which emphasizes the chronic accumulation of iron-dependent oxidative damage. By integrating bulk transcriptomic and single-cell RNA sequencing data, we systematically evaluated the potential significance of ferro-aging-related molecular features in RCC diagnosis, immune microenvironment remodeling, and tumor heterogeneity. This study not only identified candidate diagnostic biomarkers associated with ferro-aging but also characterized their biological relevance from multiple perspectives, including immune infiltration, molecular subtyping, co-expression networks, single-cell distribution, and virtual knockout analysis. Moreover, in vitro experiments preliminarily validated the role of LOX in maintaining the malignant phenotype of RCC cells, and the increased lipid peroxidation observed after LOX knockdown further supported its association with ferro-aging-related oxidative alterations.

This study has several limitations. First, although the diagnostic model showed strong discriminatory performance in the independent GSE53757 cohort, further validation in additional independent and multicenter clinical cohorts is warranted. Second, the single-cell analyses revealed heterogeneous cell-type-resolved expression patterns of the candidate core genes, while the bulk transcriptomic analyses demonstrated immune-related associations; however, these findings do not establish causal regulation of immune cell states or tumor–immune cell crosstalk. Further studies incorporating immune-cell subpopulation analysis, cell–cell communication analysis, spatial transcriptomics, and functional validation are needed to clarify the relationships between these candidate core genes and the RCC immune microenvironment. Similarly, the virtual knockout results are hypothesis-generating and require experimental validation, particularly for the predicted relationships involving NNMT and FAM134B. Third, the experimental validation was limited to LOX and did not include in vivo studies. Although LOX knockdown increased lipid peroxidation, intracellular iron accumulation, ROS production, additional ferroptosis-related markers, and cellular senescence markers were not assessed. Therefore, the present findings do not establish a direct mechanistic role of LOX in ferro-aging, and the five-gene signature should be regarded as a ferro-aging-related molecular feature rather than direct evidence of ferro-aging activation.

## Conclusion

This study identified five ferro-aging-related candidate core genes in RCC—DPEP1, LCN2, LOX, NOX4, and TLR4—with promising diagnostic potential. LOX knockdown inhibited RCC cell proliferation, migration, and invasion and increased lipid peroxidation, suggesting its potential relevance to RCC progression and ferro-aging-related processes. Further studies are needed to clarify the underlying mechanisms.

## Supporting information

S1 File Data. (XLSX)

S1 Fig ROC curve ofthe five-gene diagnostic model in the independent external validation cohort GSE53757. (PNG)

S2 Fig Correlations between candidate core gene expression and immune- and inflammation-related molecules. (PNG)

S3 Fig Cell-type-specific expression of the five candidate core genes. (PNG)

S4 Fig UMAP visualization of the expression of the five candidate core genes across major cell populations. UMAP feature plots show the expression patterns of (A) DPEP1, (B) LCN2, (C) LOX, (D) NOX4, and (E) TLR4 across seven major cell populations, including epithelial/tumor epithelial cells, myeloid cells, T cells, B cells, endothelial cells, vascular/stromal cells, and erythroid cells. The color scale indicates gene expression levels, with higher color intensity representing higher expression.
(PNG)

S5 Fig Flow cytometric analysis of lipid peroxidation in RCC cells following LOX knockdown. (A, C) Representative flow cytometry histograms of BODIPY-C11 fluorescence in ACHN (A) and 786-O (C) cells transfected with negative control siRNA (si-NC) or two independent LOX-targeting siRNAs (si-LOX#1 and si-LOX#2). A rightward shift in BODIPY-C11 fluorescence indicates increased lipid peroxidation. (B, D) Quantification of relative lipid peroxidation levels in ACHN (B) and 786-O (D) cells. Values were normalized to the corresponding si-NC group. LOX knockdown significantly increased lipid peroxidation in both RCC cell lines. ***P < 0.001; ****P < 0.0001.
(PNG)

S1 Table Detailed sample-level demographic and clinicopathological characteristics of the training cohorts (GSE40435 and GSE66272). (CSV)

S2 Table Detailed sample information for the independent external validation cohort (GSE53757). (CSV)

S3 Table List of 95 ferro-aging-related genes included in this study. (CSV)

S4 Table Primer sequences used for qRT-PCR analysis. (CSV)
